# Interactions of Rabconnectin-3 with Cav2 calcium channels

**DOI:** 10.1186/s13041-019-0483-y

**Published:** 2019-06-28

**Authors:** Maria A. Gandini, Ivana A. Souza, Jing Fan, Katherine Li, Decheng Wang, Gerald W. Zamponi

**Affiliations:** Department of Physiology and Pharmacology, Hotchkiss Brain Institute and Alberta Children’s Hospital Research Institute, Cumming School of Medicine, University of Calgary, 3330 Hospital Dr. NW, Calgary, T2N 4N1 Canada

**Keywords:** Cav2,2 calcium channels, Rabconnectin-3, Hippocampus, N-type channels, Opioid receptor

## Abstract

This study describes the interaction between Cav2 calcium channels and Rabconnectin-3, a di-subunit protein that is associated with synaptic vesicles. Immunostaining reveals that both Rabconnectin-3α (RB-3α) and Rabconnectin-3β (RB-3β) are colocalized in mouse hippocampal neurons. Co-immunoprecipitations from brain tissue is consistent with the formation of a protein complex between RB-3α and RB-3β and both Cav2.2 and the related Cav2.1 calcium channel. The coexpression of either RB-3α or RB-3β with Cav2.2 calcium channels in tsA-201 cells led to a reduction in Cav2.2 current density without any effects on the voltage-dependence of activation or inactivation. Coexpression of both Rabconnectin-3 subunits did not cause an additive effect on current densities. Finally, the presence of Rabconnectin-3 did not interfere with μ-opioid receptor mediated Gβγ modulation of Cav2.2 channels. Altogether, our findings show that Rabconnectin-3 has the propensity to regulate calcium entry mediated by Cav2.2 channels.

## Introduction

Cav2 channels are essential mediators of synaptic communication in the mammalian and invertebrate nervous systems [1–5]. Calcium entry via these channels in response to membrane depolarization leads to the release of neurotransmitters, and this process is facilitated through physical interactions of the channels with the vesicle release machinery [6–10]. Indeed, numerous synaptic proteins such as SNAP25, syntaxin 1 and synaptotagmin have been shown to bind to presynaptic calcium channels in a dynamic and often calcium-dependent manner as part of the vesicle release process [11–14]. Conversely, calcium channel activity can be substantially altered by these interactions which have been attributed to a synaptic protein interaction (a.k.a. synprint) site that is localized within the intracellular region connecting domains II and III of the pore forming Cavα1 subunit [15–18].

Rabconnectin-3 is a poorly characterized synaptic protein that is formed by α and β subunits, encoded respectively by DMXL2 and WDR7 [19–21]. Rabconnectin-3α and-3β (RB-3α and RB-3β) belong to WD40 family of proteins that contain repeats consisting of two internal dipeptide sequences, glycine–histidine and tryptophan–aspartic acid [22, 23]. RB-3α contains 12 WD40 domains and it is mainly recovered in the synaptic soluble fraction [19]. On the other hand RB-3β has 7 WD40 domains (hence the name WDR7) [20]. Very little is known about their key functional or catalytic domains, or key sites for protein-protein interactions, but it is known that WD40 domains lead to the formation of a β-propeller structure that serves as an interaction platform for a variety of proteins involved in diverse cellular processes [22, 23]. RB-3α and RB-3β are abundantly expressed in the brain where they are associated with synaptic vesicles [20]. They have been shown to interact with Rab3A GDP/GTP exchange proteins, and with members of the Rab3 small G protein family that are involved in the control of calcium dependent exocytosis [19, 20]. Although the precise role of Rabconnectin-3 in modulating exocytosis has remained enigmatic, the observation that DMXL2 haplo-insufficiency or WDR7 gene deletion cause developmental delays and mental retardation suggests a critical neurophysiological function of Rabconnectin-3 [24, 25].

Here, we present evidence that RB-3α and RB-3β form a molecular complex with Cav2.1 and Cav2.2 channels in mouse brain. Furthermore, we show that both subunits affect Cav2.2 current density, suggesting that Rabconnectin-3 may contribute to regulating brain function via alteration of presynaptic calcium influx.

## Results and discussion

**Fig. 1:**
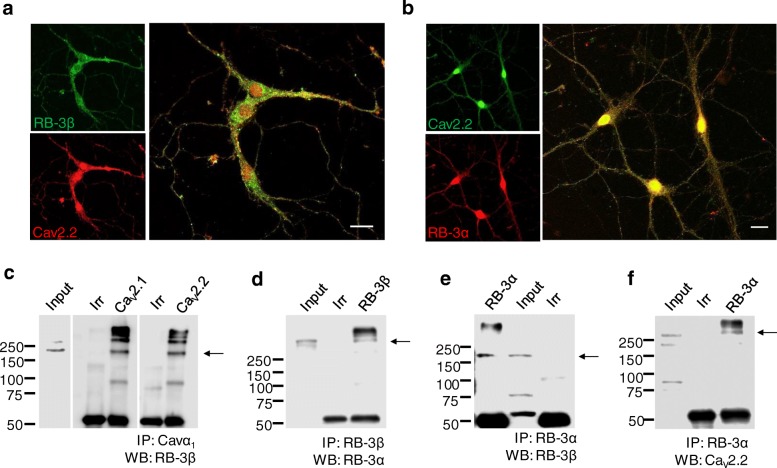
Colocalization of Rabconnectin-3 and Cav2 channels in hippocampal neurons. Confocal microscopy images from 10 DIV hippocampal neurons showing the distribution of (**a)** Cav2.2 (red) and RB-3β (green) and (**b)** Cav2.2 (green) and RB-3α (red). Scale bar 20 μm. Proteins from mouse brain were immunoprecipitated with **(c)** anti-Cav2.1or Cav2.2, **(d)** anti-RB-3β, and **(e-f)** anti-RB-3α, or with control (Irr) antibodies and followed by Western blot analysis using antibodies against the indicated proteins. The experiments are representative of 3 repetitions

We first examined the co-localization of Rabcconnectin-3 subunits with Cav2.2 calcium channels expressed endogenously in cultured mouse hippocampal neurons (10 days in vitro). Fig. 1a shows confocal images obtained from hippocampal neurons labelled with anti-RB-3β (green) and anti-Cav2.2α1 (red), revealing that both proteins are diffusely distributed in soma, axons and dendrites from neighboring cells with numerous overlapping puncta. RB-3α (red) and Cav2.2 (green) were also found to be expressed in the same neurons (Fig. 1b). Next, we performed co-immunoprecipitations between Cav2.2 channels, RB-3β and RB-3α using mouse brain homogenate. As shown in Fig. 1c, immunoprecipitation with Cav2.1 and Cav2.2 antibodies and Western blot analysis with an RB-3β antibody resulted in a band near the expected size of 163 kDa. Immunoprecipitation with an RB-3β antibody allowed us to detect RB-3α (Fig. 1d, 340 kDa band) and the reverse experiment also confirmed that the two Rabconnectin-3 subunits are in a complex (Fig. 1e). Consequently, using an RB-3α antibody, Cav2.2 channels could be immunoprecipitated (Fig. 1f). These data provide evidence that Cav2.2 channels and Rabconnectin-3 form a macromolecular protein complex, and are consistent with the immunostaining experiment.

**Fig. 2:**
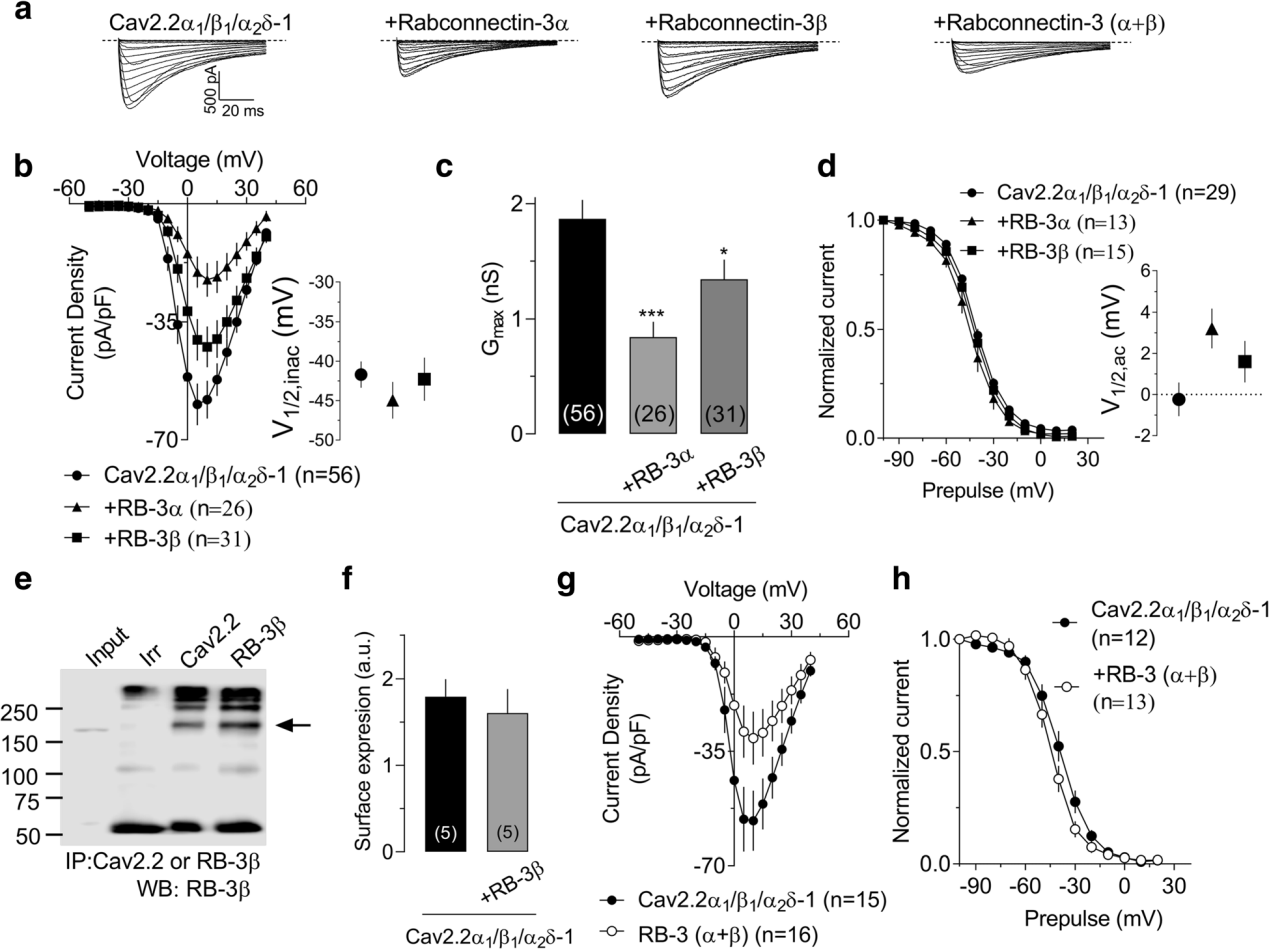
Functional effects of Rabconnectin-3 on Cav2.2 channels expressed in tsA-201 cells. (**a**) Representative set of current traces recorded in response to depolarizing steps ranging from − 50 mV to 40 mV from a holding potential of − 80 mV for cells expressing Cav2.2 channels with RB-3α or RB-3β or both. (b) Current density-voltage relationships for cells expressing Cav2.2 channels with RB-3α or RB-3β. Inset. Corresponding mean half activation potential. (c) Corresponding maximal conductance. Numbers shown in the bars reflect numbers of cells (d) Steady-state inactivation curves for cells expressing Cav2.2 channels with RB-3α or RB-3β. Inset. Corresponding mean half activation potential. (e) Proteins from tsA-201 cells transfected with Cav2.2α_1_ and RB-3β were immunoprecipitated with anti-Cav2.2, anti-RB-3β or control (Irr) antibodies and followed by Western blot analysis using anti-RB-3β. (f) Quantification of biotinylation experiments from tsA-201 cells transfected with Cav2.2/β_1_/α_2_δ-1 with and without RB-3β. Biotinylated cell surface protein was isolated and normalized to Na/K-ATPase levels. Numbers shown in the bars reflects numbers of independent experiments. (g) Current density-voltage relationships for cells expressing Cav2.2 channels with RB-3(α + β). (h) Steady-state inactivation curves for cells expressing Cav2.2 channels with RB-3(α + β).

To ascertain whether Rabconnectin-3 can alter Cav2.2 channel function, we recorded whole cell barium currents through recombinant Cav2.2 (+Cavβ_1b_ + Cavα_2_δ-1) channels expressed in tsA-201 cells in the absence or presence of Rabconnectin-3. Representative whole-cell Cav2.2 current recordings are shown in Fig. 2a. Figure 2b shows the average current density-voltage relationships (peak current amplitude normalized by Cm) in response to membrane depolarizations from a holding potential (Vh) of − 80 mV. The expression of either RB-3α or RB-3β significantly reduced the current density across a wide range of voltages and this is also reflected in a decrease in Gmax (Fig. 2c) We did not observe changes in voltage for half-maximal activation (Fig. 2b inset) and the slope factor (not shown). Neither Rabconectin-3 subunit had any significant effect on half-inactivation potential (Fig. 2d). Rabconnectin-3 is a cytoplasmic protein that, as we show here, appears to interact with Cav2.2 channels. When we transfected just the pore forming Cav2.2 α_1_ subunit with RB-3β and performed a co-immunoprecipitation we found an interaction between these proteins (Fig. 2e). This means that the ancillary Cavβ subunit is not necessary for forming the molecular complex. Interestingly, biotinylation experiments from cells expressing RB-3β and Cav2.2 channels did not reveal any difference in cell surface expression (Fig. 2f), suggesting that the decrease in current density is not due a reduction in the number of channels at the plasma membrane. Coexpression of both Rabconnectin-3 subunits produced effects that were similar to those observed in the presence of RB-3α (Fig. 2g and h), indicating that there are no synergistic effects (current density at 10 mV in pA/pF - Cav2.2/β_1_/α_2_δ-1: control − 58.06 ± 5.6; +RB-3α: 22.02 ± 5.0; +RB-3β: -42.35 ± 6.1; Cav2.2/β_1_/α_2_δ-1: control − 56.27 ± 9.0; +RB-3(α + β): -30.95 ± 8.1; *P* > 0.05 compared to RB-3α), and that the formation of a complete Rabconnectin-3 complex is still capable of modulating Cav2.2 current density.

**Fig. 3:**
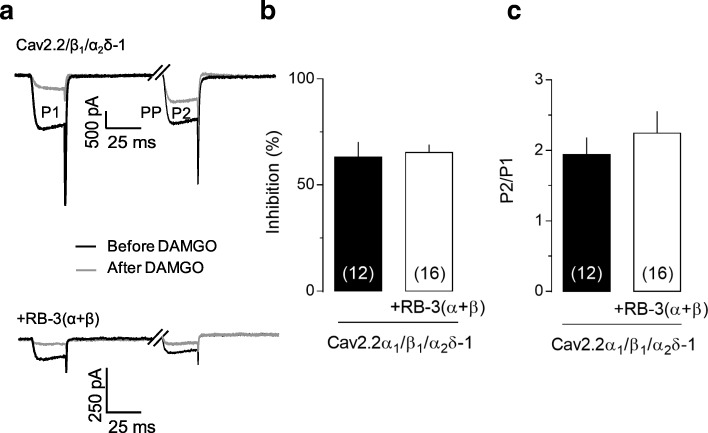
Lack of effect of Rabconnectin-3 on G protein modulation of Cav2.2. (**a**) Representative set of Cav2.2 currents recorded before or after the application of 10 μM DAMGO. As noted in the Methods section, the first current in each trace is evoked by a test depolarization to + 10 mV (P1), the second inward current in a given trace is evoked by a 10 mV test depolarization (P2) that is preceded by a strong depolarizing prepulse (PP, note that the prepulse-evoked outward current has been blanked out). The increase in current amplitude seen during P2 in the presence of DAMGO reflects prepulse relief of Gβγ modulation. (b) Percentage of peak current inhibition (during P1) of Cav2.2 currents after application of 10 μM DAMGO in the presence of absence of RB-3(α + β). (c) Voltage dependent pre-pulse facilitation measured in the presence of DAMGO. The bars reflect the current evoked by test pulse P2 normalized to the current evoked by test pulse P1. This experiment was performed either in the presence or the absence of RB-3(α + β). Numbers in parentheses reflect numbers of cells.

Given the bulky nature of Rabconnectin-3, we wondered if its presence might affect regulation of the channels by G protein βγ subunits which are known to target the N-terminus and domain I-II linker regions of Cav2.2 [26–30]. This was tested by coexpressing μ-opioid receptors with the channel along with RB-3α plus RB-3β, and then activating the receptors via DAMGO application (Fig. 3a). We found no difference in receptor mediated Cav2.2 channel inhibition (Fig. 3b), nor in the relief of this inhibition via strong voltage pulses (Fig. 3c). Hence, G proteins retain access to their binding site on the Cav2.2 channels, suggesting that the Rabconnectin-3 interaction site is distinct from that targeted by Gβγ.

At this point we do not know where on the channel the Rabconnectin-3 interaction site is located, and whether the interaction is direct, or mediated via an adaptor protein. It is interesting to note that both Rabconnectin-3 subunits independently produced a reduction in current density, which may suggest that there are two separate interaction sites for these two subunits, or that they both target the same region, and in such a manner that the functional effects are preserved when both subunits are present. It is interesting to note that RB-3β was less effective in reducing current densities compared to RB-3α, but perhaps not unexpected given that these two proteins show considerable differences in their numbers of WD40 repeats. We also do not know whether the reduction in current density is due a reduction in single channel amplitude or in maximum open probability. It will also be interesting to determine whether Rabconnectin-3 can interfere with the modulation of channel activity by synaptic proteins targeting the synprint site [7, 8, 31, 32].

We reiterate that the importance of a fully functional Rabconnectin-3 protein is important for brain health. Tata and colleagues [25] described a case of three brothers with an in-frame deletion in the RB-3α encoding gene (DMXL2). This resulted in reduced RB-3α mRNA levels and manifested itself as a series of neurodevelopmental disorders such as mental retardation, dysregulation of the hypothalamus-pituitary hormonal axis and metabolic disorders such as diabetes and hypoglycemia. On the other hand, it has been shown that low DMXL2 expression in mice causes abnormal dendritic development. DMXL2+/− mice show differences in corpus callosum neuroanatomy compared to wild type animals [25]. Along these lines, Chen and co-workers [33] linked an RB-3α missense variant to nonsyndromic hearing loss. Altogether, this strongly suggests that RB-3α is a key regulator in neuronal and homeostatic processes. RB-3β is deleted along with the TXNL1 gene in a condition termed 18q syndrome, a condition characterized by mental retardation that varies in severity from patient to patient, along with a wide array of anatomical and physiological abnormalities, including dystonia [24, 34]. It is unlikely that all these phenotypic abnormalities are mediated through alterations of Cav2.2 channel activity. That being said, our experiments in tsA-201 cells would be consistent with a gain of Cav2.2 channel function. In this context, it is interesting to note that a gain of Cav2.2 channel function through a missense mutation has been associated with dystonia [35].

In summary, we present the first description of a physical and functional interaction between Rabconnectin-3 and Cav2.2 calcium channels. This interaction adds to a list of many synaptic proteins that associate with this important presynaptic calcium channel.

## Materials and methods

### cDNAs

Wild type rat calcium channel subunit cDNAs were donated by Dr. Terry Snutch (University of British Columbia, Vancouver, BC) and subcloned into pcDNA3.1 vectors. Human WDR7 cDNA was purchased from Thermo Scientific and human DMXL2 cDNA was obtained from Origene™.

### cDNA transfection

After splitting and seeding to 50% confluence, tsA-201 cells were transfected with 3 μg of each plasmid encoding Cav2.2α1, Cavβ1 and Cavα2δ-1, respectively, in the presence or absence of WDR7, DMXL2 or both cDNAs. In addition, 0.5 μg of cDNA encoding green fluorescent protein was added to the transfection mixture to identify and select transfected cells. For G protein studies 3 μg of the μ-opioid receptor cDNA were added. Cells used for electrophysiology experiments were moved to 30 °C after transfection, whereas those used for Western blotting were maintained at 37 °C.

### Patch clamp recordings

Electrophysiological recordings were performed using whole cell patch-clamp at room temperature (22–24 °C). Currents were recorded using an Axopatch 200B amplifier linked to a computer with pCLAMP9.2 software. The external recording solution contained (in mM): 5 BaCl_2_, 10 TEA-Cl, 1 MgCl, 128 NaCl, 5 KCl, 10 HEPES and 10 glucose (pH 7.4). Patch pipettes were filled with a solution containing (in mM) 110 CsCl, 2.5 MgCl, 10 EGTA, 10 HEPES, 3 ATP, and 0.5 GTP (pH 7.4). Current densities were obtained by dividing peak current by the whole cell capacitance. Current density–voltage relationships were generated from the peak current obtained during 250 ms pulses between − 50 to + 40 in 5 mV increments from a holding potential of − 80 mV. Steady-state inactivation was measured by applying 2 s conditioning pulses from − 100 to + 20 mV in 10 mV increments followed by a 100-ms test pulse to + 10 mV. I-V relationships were fitted with a Boltzmann equation of the form: I = Gmax*(Vm − Vr)/(1 + exp.(−(Vm − V1/2act)/k)), where I is the peak current, Vm is the membrane voltage, V1/2act is the half activation potential, Vr is the reversal potential, and k is the slope factor. Steady-state inactivation curves were fitted with the equation: I/Imax = 1/(1 + exp.((Vp − V1/2inac)/k)), where I/I max is the normalized peak current, Vp is the conditioning pre-pulse, V1/2inac is the half-inactivation potential and k is the slope factor.

G protein modulation induced by μ-opioid receptor activation was assessed as described by us previously [36]. In brief, the extent of total G protein inhibition was assessed by a multi pulse protocol in which cells are held at − 80 mV. Currents are evoked by a 25 ms test depolarization to + 10 mV (P1), followed by a 500 ms repolarization to − 80 mV. Subsequently, a strong 50 ms depolarizing pulse to + 100 mV is applied (which causes a reversal of Gβγ mediated voltage-dependent inhibition, see [37]). This is followed by 5 ms repolarization and then a second test pulse to + 10 mV (P2). Total G protein inhibition is determined by monitoring the DAMGO-induced reduction in peak current amplitude during P1. Voltage-dependent G protein modulation is determined by the extent of prepulse relief of inhibition as assessed by calculating the ratio of current amplitudes observed during P2 and P1 in the presence of receptor agonist.

### Co-immunoprecipitation assays and western blots

Cells were detached from culture dishes, washed with phosphate-buffered saline, and lysed in single-detergent lysis buffer (50 mM Tris–Cl, 150 mM NaC’l, 1% Triton X-100, and Complete 1×; Roche Applied Science). Protein concentration was determined using the Bradford assay. Rat brain proteins were lysed in RIPA buffer (25 mM Tris–HCl (pH 7.6), 150 mM NaCl, 1% NP-40, 1% sodium deoxycholate, 0.1% SDS, and Complete 1×). Fifty micrograms of protein samples were boiled for 5 min in protein-loading buffer (1.7% SDS, 0.1 M 2-mercaptoethanol, 5% glycerol, 58 mM Tris–Cl, and 0.002% bromophenol blue, pH 6.8). Co-immunoprecipitation assays were performed using rat brain proteins or lysates from transfected tsA-293 cells. One milligram of proteins was incubated with 3 μg of specific or irrelevant (as an isotype control) antibodies. Immune complexes were resolved in 8–15% SDS-polyacrylamide gels and transferred to nitrocellulose membranes then immunoblotted using the following antibodies: Cav2.1(1:500; Alomone ACC-001), Cav2.2 (1:500; Alomone ACC-002), WDR7 (1:1000; Santa Cruz sc-85,210), and DMXL2 (1:500; Novus Biological NBP1–93618).

### Cell surface biotinylation

Transfected cells were washed with ice-cold HEPES-based saline solution (HBSS) and incubated on ice for 15 min to stop trafficking of proteins. Surface proteins were biotinylated for 1 h on ice with 1 mg/ml of EZ- Link Sulfo-NHS-SS-Biotin (Thermo Scientific). The reaction was quenched with 100 mM glycine for 15 min, and cells were lysed in modified RIPA buffer (in mM: 50 Tris, 150 NaCl, 5 EDTA, 1% Triton X-100, 1% NP-40, 0.2% SDS, pH 7.4) for 45 min. Protein quantification was performed using a Bio-Rad protein assay dye, and 2 mg of lysates was incubated with 100 μl of Neutravidin beads (Thermo Scientific) for 1.5 h at 4 °C. Beads were washed three times with lysis buffer and proteins eluted with 2× Laemmli sample buffer. Biotinylated proteins and lysates were resolved by SDS-PAGE and analyzed by western blot using anti-Cav2.2 (1:500, ACC-002 Alomone) and 1/5000 anti-Na/K-ATPase (1:5000, AB 7671 Abcam) antibodies. For each experiment, background was subtracted and integrated density of bands was measured and normalized to the Na/K-ATPase signal as loading control.

### Immunostaining

Hippocampal neurons (10 DIV) were blocked for 1 h with 0.5% BSA + 10% NGS + 0.3% Triton X-100 in 1X PBS. Sections were then incubated with the following antibodies: Cav2.2 (1:100; Alomone ACC-002 or 1:100; Synaptic systems 152,311), WDR7 (1:100; Santa Cruz sc-85,210) and DMXL2 (1:100, Santa Cruz sc-162,739) overnight at 4 °C. Neurons were washed 3 times with PBS before incubation with the following secondary antibodies: anti-rabbit (Alexa Fluor 488, 1:1000, Invitrogen A21206), anti-mouse (Alexa Fluor 633, 1:500, Invitrogen A21050) and anti-goat (Alexa Fluor 633, 1:1000, Invitrogen A11055) for 1 h at room temperature. Images were taken using a Zeiss LSM 510 confocal microscope.

### Statistics

All error bars reflect standard errors. Statistical analysis was conducted with Student’s t-tests. Significance was set at 0.05. Asterisks denote significance as follows: * *p* < 0.05, ** *p* < 0.01, ***p < 0.001.

## Data Availability

The data used in our study are available from the authors on reasonable request.

## Abbreviations

RB-3: Rabconnectin-3
SNAP25: Synaptosome Associated Protein 25
WD: Tryptophan-aspartic acid
